# Supplementary material for: Improving Soluble Expression of SARS-CoV-2 Spike Priming Protease TMPRSS2 with an Artificial Fusing Protein
Source: Int J Mol Sci. 2023 Jun 22;24(13):10475. doi: 10.3390/ijms241310475 (PMC10341623; doi:10.3390/ijms241310475)
Supplement: Supplementary file 1 [file ijms-24-10475-s001.zip › ijms-2421991-supplementary.pdf]

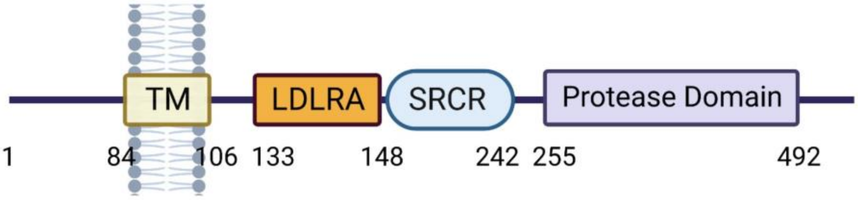

Supplementary Figure S1. Schematic representation of multi-domain protein TMPRSS2.TM: transmembrane domain; LDLRA: low-density lipoprotein receptor A domain; SRCR: scavenger receptor cysteine-rich domain; Protease Domain: serine protease domain.

Plasmid	Inserted gene	Vector	Selection marker	Fusion tag
pET-His-TMPRSS2	His-TMPRSS2	pET-28	Kan	None
pET-MBP-TMPRSS2	MBP-TMPRSS2	pET-21a (+)	Amp	MBP
pET-SUMO-TMPRSS2	SUMO-TMPRSS2	pET-28	Kan	SUMO
pGEX-GST-TMPRSS2	GST-TMPRSS2	pGEX-6P-1	Amp	GST
pET-XXA-TMPRSS2	His-XXA-TMPRSS2	pET-21a (+)	Amp	XXA-His

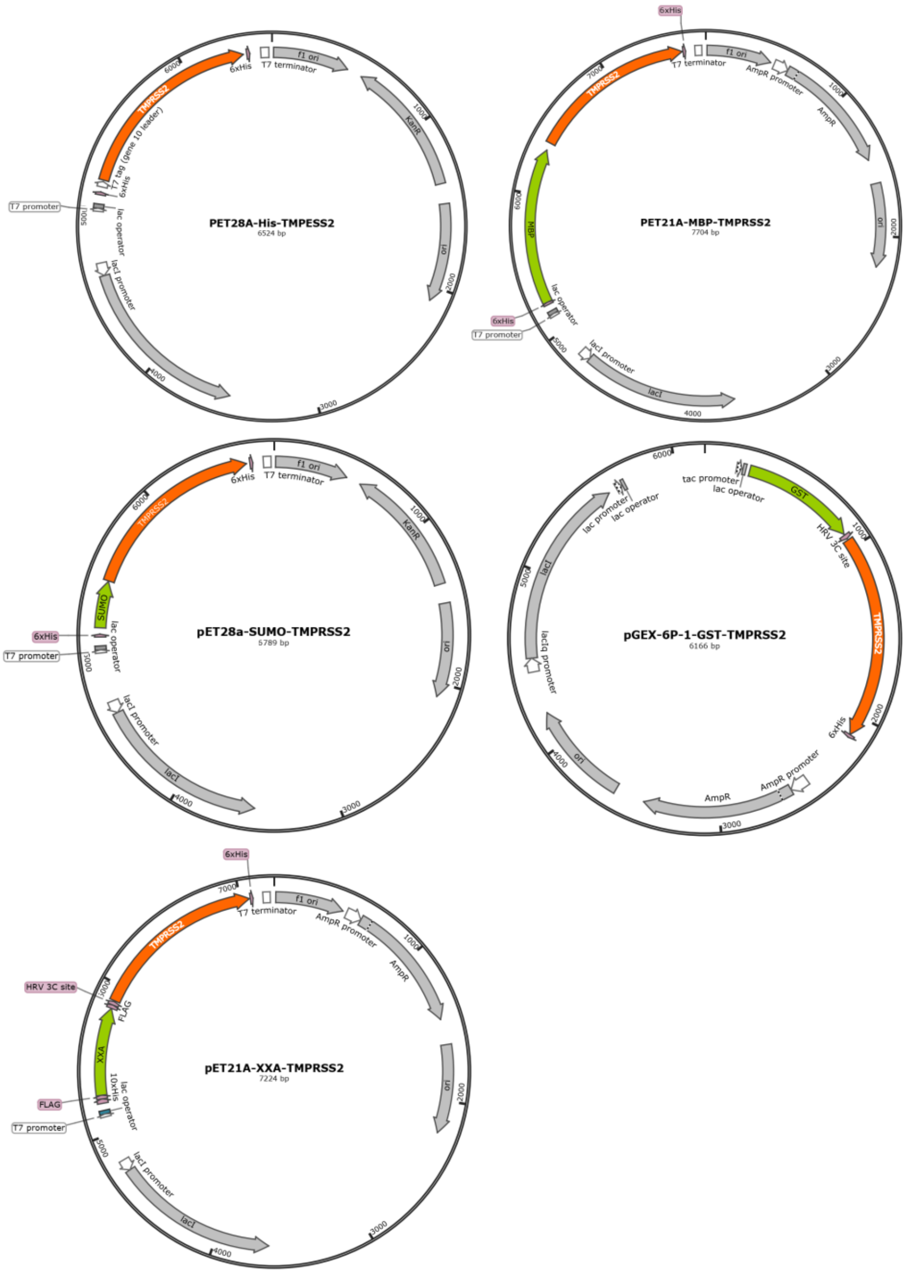

Supplementary Figure S2. Recombinant plasmids constructed in this study. Schematic diagram of different recombinant plasmids expressing His-TMPRSS2, MBP-TMPRSS2, SUMO-TMPRSS2, GST-TMPRSS2 and XXA-TMRPSS2.

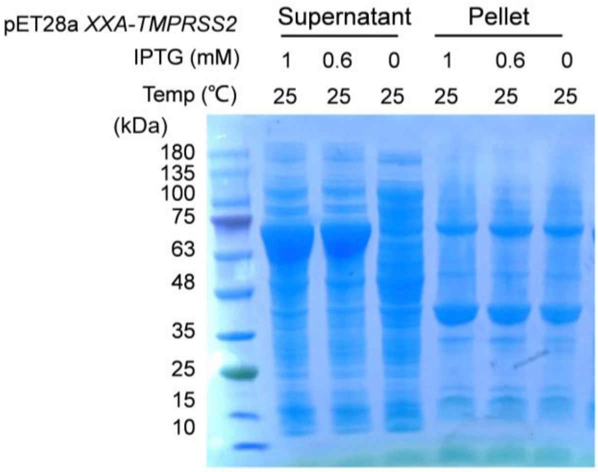

Supplementary Figure S3. XXA-TMPRSS2 was induced at different condition. *E. coli* cells transfected with pET-28a-XXA-TMPRSS2 were induced with 0.6 or 1 mM IPTG at 25 °C for 16 hours, and the soluble supernatant and insoluble pellet fractions were analyzed separately.

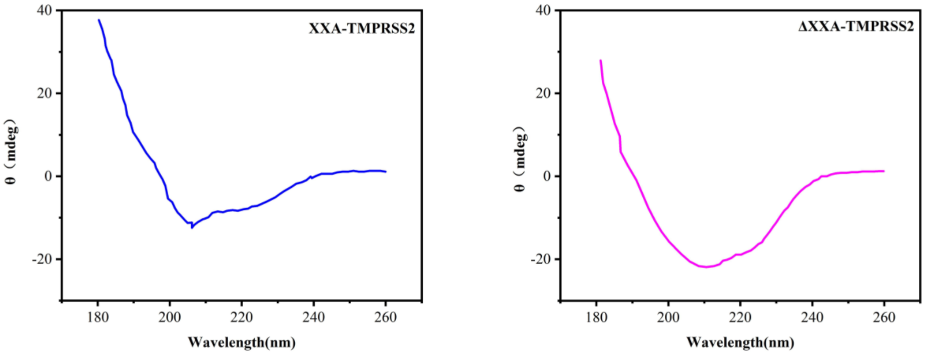

Supplementary Figure S4. CD spectrum of XXA-TMPRSS2 and ΔXXA -TMPRSS. The CD spectrum of XXA-TMPRSS2 and ΔXXA -TMPRSS protein were captured using a spectrophotometer over the entire UV wavelength range (180-260 nm) at 20 °C.

Name	Sequence
AXX	MQDELADAKSAIEAKHAVSDAAKKETVTGAAADVQEARDVTQDQRQNLGYAEQK<br>AADTLGDVAAAQEAYESAKQRASEAAEGAKSTASELGGSAERAVRDAAGGAEGAGR<br>DAQGAAREGLKGAEGAGATDEARRHAEDVADTAKEKYSELKGDAKEGLGRAQAKG<br>EDLAGDASKAAQDAADRLK
XXA	KLRDAADQAAKSADGALDEGKAQARGLGEKADGKLESYKEKATDAVEAHRRAEDA<br>AEAGKLGERAAGQADRGAGEAGGAADRVAREASGGLESATSKAGEAAESARQKAE<br>YAEQAAAKDGLTAAKQEAYGLNQRQDQTVDRATEQVDAAAGTVTEKVKQAADSV<br>AHKATEIASKAKDALSEDQM

Supplementary Table S1. Amino acid sequences of antifreeze protein AXX and artificial protein XXA.
